# Exploring microRNA-Mediated Immune Responses to Soil-Transmitted Helminth and Herpes Simplex Virus Type 2 Co-Infections

**DOI:** 10.3390/diseases13010006

**Published:** 2025-01-01

**Authors:** Roxanne Pillay, Pragalathan Naidoo, Zilungile L. Mkhize-Kwitshana

**Affiliations:** 1 Department of Biomedical Sciences, Faculty of Natural Sciences, Mangosuthu University of Technology, Umlazi, Durban 4031, South Africa; 2 Department of Medical Microbiology, College of Health Sciences, School of Laboratory Medicine & Medical Sciences, Nelson R. Mandela School of Medicine, University of KwaZulu-Natal, Durban 4001, South Africa; 3 Division of Research Capacity Development, South African Medical Research Council (SAMRC), Tygerberg, Cape Town 7505, South Africa; 4 Biomedical Sciences Department of Life and Consumer Sciences, College of Agriculture and Environmental Sciences, University of South Africa, Florida Campus, Johannesburg 1710, South Africa

**Keywords:** microRNAs, soil-transmitted helminths, herpes simplex virus-2, co-infection, immunological interactions, host immunity, immunomodulation

## Abstract

Over the last two decades, the field of microRNA (miRNA) research has grown significantly. MiRNAs are a class of short, single-stranded, non-coding RNAs that regulate gene expression post-transcriptionally. Thereby, miRNAs regulate various essential biological processes including immunity. Dysregulated miRNAs are associated with various infectious and non-infectious diseases. Recently co-infection with soil-transmitted helminths (STHs) and herpes simplex virus type 2 (HSV-2) has become a focus of study. Both pathogens can profoundly influence host immunity, particularly in under-resourced and co-endemic regions. It is well known that STHs induce immunomodulatory responses that have bystander effects on unrelated conditions. Typically, STHs induce T-helper 2 (Th2) and immunomodulatory responses, which may dampen the proinflammatory T-helper 1 (Th1) immune responses triggered by HSV-2. However, the extent to which STH co-infection influences the host immune response to HSV-2 is not well understood. Moreover, little is known about how miRNAs shape the immune response to STH/HSV-2 co-infection. In this article, we explore the potential influence that STH co-infection may have on host immunity to HSV-2. Because STH and HSV-2 infections are widespread and disproportionately affect vulnerable and impoverished countries, it is important to consider how STHs may impact HSV-2 immunity. Specifically, we explore how miRNAs contribute to both helminth and HSV-2 infections and discuss how miRNAs may mediate STH/HSV-2 co-infections. Insight into miRNA-mediated immune responses may further improve our understanding of the potential impact of STH/HSV-2 co-infections.

## 1. Introduction

MiRNAs are a class of small, single-stranded, non-coding RNA molecules known to regulate gene expression at a post-transcriptional level. By binding to their target mRNAs, miRNAs regulate a wide range of essential physiological functions, including immunity. Moreover, dysregulated miRNA expression is a hallmark of various human infections and disease processes. In this way, miRNAs may serve as potential biomarkers in the diagnosis and prognosis of various infections and as novel therapeutic targets [1].

Soil-transmitted helminths (STHs) are among the most prevalent parasitic infections. Globally more than 1.5 billion humans are infected, particularly those living in under-resourced tropical and subtropical countries across sub-Saharan Africa, the Americas, China, and East Asia. Limited access to clean water, proper sanitation, and healthcare facilities exacerbates the risk and transmission of infection. The three major groups of STHs are roundworms (*Ascaris lumbricoides*), whipworms (*Trichuris trichiura*), and hookworms (*Necator americanus* and *Ancylostoma duodenale*) [2].

A significant epidemiological feature of under-resourced countries is their high burden of sexually transmitted viral infections including herpes simplex virus type II (HSV-2), which causes genital herpes [3]. In 2016, more than 491.5 million people (13.2%) worldwide, aged between 15 and 49 years, were reportedly infected with HSV-2; most infections were reported in the WHO African region [4].

It is well recognised that STHs stimulate dominant Th2 immune responses that downregulate Th1 and Th17 immune responses. Moreover, they possess potent strategies to modulate host immunity to unrelated infections [5]. By employing the following strategies, STH modulate immunity: (i) they induce host resistance and tolerance responses, (ii) they secrete immunomodulatory products such as excretory-secretory products (ESP), and (iii) they interact with the intestinal microbiome [6]. HSV-2 infection, on the other hand, triggers a proinflammatory Th1 immune response [7].

The immunomodulatory capabilities of STHs have been shown influence the outcomes of concomitant infections, which occur commonly in under-resourced countries [8]. Moreover, STH co-infections have been shown to influence immune responses to sexually transmitted viruses, including human papillomavirus (HPV) [9,10], human immunodeficiency virus (HIV) [11,12,13], and HSV-2 [14]. Given the overlapping incidences of STH and HSV-2 infections in under-resourced countries [15], the likelihood of STH/HSV-2 co-infections is high, with potential implications for host immunity. Because STHs are associated with Th2 and immunomodulatory responses, their immunomodulatory effects may potentially dampen the Th1 responses triggered by HSV-2. Surprisingly, very little is known about the epidemiology of STH/HSV-2 co-infections or the immunological interactions between these pathogens. Moreover, how miRNAs may contribute to these potential immunological interactions is undetermined. This dearth of information highlights a significant gap in our understanding of the impact of STH/HSV-2 co-infections in under-resourced countries. Exploring the role of miRNAs in immune responses to STH and HSV-2 single and co-infections could provide valuable insight into various key aspects of host immunity.

In this article, we consider how STH co-infection may suppress essential anti-HSV-2 immune responses, thereby potentially exacerbating HSV-2 severity. Specifically, the potential role of miRNAs in shaping immune responses to STH/HSV-2 co-infection is explored. We highlight the need for studies that focus on STH/HSV-2 co-infections and discuss future work needed to elucidate the role of miRNAs in immune responses elicited by these pathogens during co-infection. Such work may contribute to alleviating the burden of STHs and HSV-2 infections in under-resourced countries.

## 2. Overview of Soil-Transmitted Helminth Infections and Host Immune Responses

Helminths are widely distributed parasitic worms that compose of two major phyla: the Nematoda (roundworms) and Platyhelminths (flatworms). STHs belong to Nematoda, while Platyhelminths are divided into two classes, cestodes (tapeworms) and trematodes (flukes) [16,17,18,19]. In this article, we have focussed on STHs.

Infections with STHs, also known as intestinal helminths, are among the most prevalent helminth infections, worldwide [2]. The four most prevalent STH species are *Ascaris lumbricoides* (roundworms), *Trichuris trichiura* (whipworm), and *Ancylostoma duodenale* and *Necator americanus* (hookworms). These infections disproportionately affect vulnerable and impoverished countries, where access to clean water, proper sanitation, and healthcare facilities is limited [2,20]. While most infections are mild and asymptomatic, STHs are associated with significant morbidity due to the chronicity of infections. Severe infections are associated with malnutrition, anaemia, and impaired physical and mental growth [19,20,21].

STHs are primarily transmitted via faecal contamination of the environment and food sources in areas that lack clean water and sanitation facilities. Infection occurs following ingestion of embryonated eggs from contaminated water or food sources (*Ascaris lumbricoides* and *Trichuris trichiura)* or penetration of the skin by infective larvae (*Necator americanus* and *Ancylostoma duodenale)*. STHs have complex lifecycles comprising various developmental stages, including migration of larvae through host tissues, maturation into adult worms, reproduction, and faecal excretion of eggs into the environment via an infected host [19,20,21].

STHs have coevolved with their hosts over many centuries, consequently developing several mechanisms to undermine host immunity. Regardless of their routes of infection and lifecycles, STHs typically elicit a dominant Th2 immune response in their hosts. STH-induced Th2 immune responses control inflammation, promote repair of damaged tissue, and support host tolerance. In this way, STHs establish long-standing and insidious infections with low host mortality but high morbidity [6,22,23]. A STH-driven Th2 phenotype is initiated by alarmin cytokines interleukin (IL)-25 and IL-33 and thymic stromal lymphopoietin (TSLP). Several co-ordinated immunological and physiological responses follow, driven by the release of Th2 cytokines (IL-4, IL-5, IL-9, and IL-13). There is an amplification of alternatively activated macrophages, increased levels of parasite-specific immunoglobulin (Ig), non-specific IgE, eosinophils, basophils, and degranulation of mast cells. Smooth muscle contraction and goblet cell hyperplasia contribute to the “weep and sweep” mechanism required for STH expulsion. STH-induced Th2 immune responses typically downregulate Th1 and Th17 inflammatory cytokines. Additionally, in chronic STH infections, anti-inflammatory cytokines [IL-10 and transforming growth factor-β (TGF-β)] and regulatory cell populations (forkhead box P3 (FoxP3)-expressing regulatory T cells and B cells) are produced, thus creating an environment that favours parasite survival [6,23,24,25]. STH infections rarely occur in isolation, particularly in under-resourced regions, and co-infections with bacterial, protozoan, and viral pathogens occur commonly. Consequently, STH-induced immunomodulation has bystander effects on host immune responses to a range of pathogens, as well as allergies, autoimmune diseases, and inflammatory conditions, with subsequent beneficial or detrimental outcomes for co-infected hosts [5,6,8].

## 3. Overview of HSV-2 Infection and Host Immune Responses

HSV-2 is a double-stranded human DNA virus and is a member of the Herpesviridae family and alpha-herpesvirus subfamily [26].

HSV-2 infects keratinocytes that line genital mucosa, leading to genital lesions [27]. Infections are primarily self-limiting and asymptomatic but may cause life-threatening complications in newborns and immunocompromised individuals. HSV-2 causes lifelong infections due to its ability to establish latency in sensory neurons and ganglia [28]. Reactivation of genital lesions occurs commonly in symptomatic individuals and viral shedding by asymptomatic and symptomatic individuals facilitating the spread of infection [7]. Importantly, HSV-2 can promote the acquisition of other sexually transmitted infections and is considered a key driver of HIV transmission [4,29]. Thus, HSV-2 represents a significant global public health concern.

The anti-HSV-2 immune response is complex, involving both the innate and adaptive components of immunity. Innate immunity is vital for the initial control of the virus and mediates an effective adaptive immune response. Following primary HSV-2 infection, the toll-like receptor (TLR) signalling pathway is stimulated and produces type 1 interferon (IFN), mainly IFNα and IFNβ. Type 1 IFNs activate innate cells such as natural killer cells and plasmacytoid dendritic cells and stimulate production of Th1 inflammatory cytokines IL-1, IL-6, and tumour necrosis factor-α (TNF-α). Natural killer cells also stimulate cytokine production and kill virally infected cells, and plasmacytoid dendritic cells produce type 1 IFN. Adaptive immunity against HSV-2 comprises humoral and cellular responses. In particular, CD4^+^ and CD8^+^ T cells are important for clearing infection through the production of IFNγ [30,31]. However, the virus has evolved strategies to evade host immunity and establishes latency in sensory neurons and ganglia, thereby causing lifelong infections [28]. Currently there are no vaccines for HSV-2. The mainstay drugs used to treat HSV-2 infection include acyclovir, valacyclovir, and famciclovir. However, these drugs are ineffective in eliminating viral shedding and are associated with side effects and limited bioavailability [32]. Drug resistance to antivirals has also been reported [33]. Therefore, new and more effective therapeutic approaches are needed.

## 4. Soil-Transmitted Helminth and HSV-2 Co-Infection: A Potential Role for miRNAs

Recently, the interaction between STH and HSV-2 has become a focus of study. In their landmark study, Chetty et al. (2021) demonstrated that acute infection with *Nippostrongylus brasiliensis,* a murine hookworm with a similar lifecycle to the human hookworms *Ancylostoma duodenale* and *Necator americanus*, systemically enhanced vaginal pathology in mice co-infected with HSV-2. Vaginal pathology was associated with an IL-5-driven Th2 response that resulted in accumulation of eosinophils and enhanced ulceration in the vaginal tracts of co-infected mice [14]. Their findings suggest that STH co-infection may potentially impair anti-HSV-2 immune responses and exacerbate HSV-2 pathology, thus negatively impacting female reproductive health. Given the geographical overlap of STH and HSV-2, it is plausible that co-infections occur in individuals living in co-endemic regions, with subsequent consequences for HSV-2 pathology. STH co-infection could influence the outcome of HSV-2 by inducing Th2 responses that modulate anti-HSV-2 Th1 responses. No epidemiological evidence has been reported; however, it remains possible that STH/HSV-2 co-infections may contribute to altered pathology, immune responses, or transmission dynamics, highlighting the need for focussed studies that investigate their clinical and public health implications.

While we postulate that STH/HSV-2 co-infection may exacerbate HSV-2 pathology, it is important to consider other key role players that may contribute to host immune responses and infection outcomes. In this regard, appropriate host immune responses to either pathogen requires the co-ordinated control of specific genes. Recently, the central role of miRNAs in regulating immune responses to various infectious and non-infectious diseases has been recognised [34,35,36]. In this way, miRNAs may regulate important aspects of host immunity to STH/HSV-2 co-infection.

## 5. MiRNA Biogenesis

MiRNAs are a class of small, non-coding, single-stranded RNAs, of approximately 22 nucleotides in length. They regulate the expression of their target mRNAs at a post-transcriptional level [1]. The lin-4 and let-7 miRNAs were first described in 1993 in *Caenorhabditis elegans* and were shown to be essential for normal development of the nematode [37]. By regulating the expression of their target mRNAs, miRNAs participate in various essential biological processes including normal development, differentiation, metabolism, and immunity. Moreover, miRNAs have been identified in a diverse range of pathogens, including STHs [38,39,40,41,42,43] and HSV-2 [44,45,46,47,48]. Dysregulated miRNA expression is a hallmark of various infections and disease processes. Thus, miRNAs may serve as key biomarkers in the diagnosis and prognosis of various conditions and may potentially serve as novel therapeutic targets [1].

**Figure 1:**
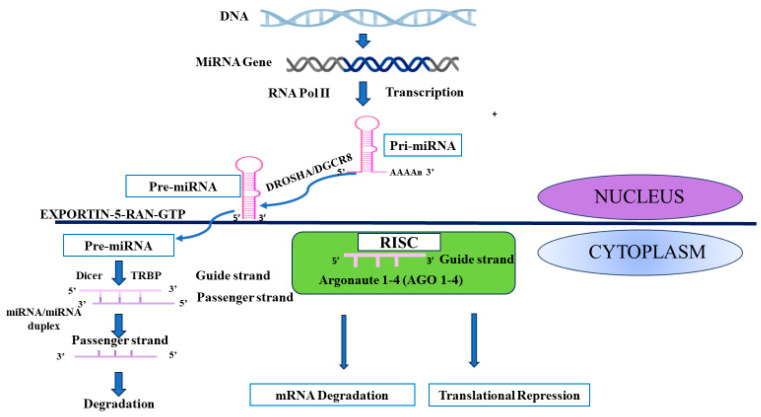
Schematic diagram of the biogenesis of miRNAs via the canonical pathway from the transcription of pri-miRNAs to the translational repression/mRNA degradation. Sequential steps are described in text.

The biogenesis of miRNAs has been comprehensively reviewed elsewhere [1,49]. Briefly, miRNA biogenesis is a complex process involving several sequential steps that result in mature, functional miRNAs. Biogenesis may occur via one of two pathways: the canonical and non-canonical pathways. The canonical pathway forms the main pathway by which miRNAs are synthesized (Figure 1). It begins in the nucleus where miRNA genes are transcribed by RNA polymerase II to form an initial transcript, called the primary miRNA (pri-miRNA). A pri-miRNA, which is typically several hundred nucleotides long, forms a hairpin structure and is usually capped and polyadenylated. The pri-miRNA is then recognized and processed in the nucleus by the Drosha-DGCR8 (DiGeorge syndrome critical region 8) complex. Here, Drosha cleaves the pri-miRNA near the base of the hairpin, forming a smaller hairpin structure called the precursor miRNA (pre-miRNA). The pre-miRNA is then exported from the nucleus to the cytoplasm via the exportin-5/Ran-GTP complex. Further processing of the pre-miRNA occurs in the cytoplasm, where the enzyme Dicer cleaves the pre-miRNA near its hairpin loop to form a double-stranded miRNA/miRNA duplex [1].

The non-canonical and canonical pathways differ by one or more steps. Specifically, the enzymes Drosha and DGCR8 are essential for miRNA biogenesis to occur via the canonical pathway. Therefore, in their absence, miRNA biogenesis follows the non-canonical pathway. Several non-canonical miRNA biogenesis pathways have been described [49]. Briefly, non-canonical miRNA biogenesis comprises Drosha/DGCR8-independent and Dicer-independent pathways. In the Drosha/DGCR8-independent pathway, pre-miRNAs resemble Dicer substrates. Examples of Drosha/DGCR8-independent pathway include the following: (i) mirtrons, which are formed from mRNA introns during splicing and (ii) 7-methylguanosine (m7G)-capped pre-miRNAs, which are exported directly to the cytoplasm via exportin 1, without Drosha cleavage. In contrast, Dicer-independent miRNAs are processed by Drosha from endogenous short hairpin RNA (shRNA) transcripts. These pre-miRNAs need AGO2 to mature in the cytoplasm, as they are much shorter than Dicer substrates. Pre-miRNAs are then loaded onto AGO2, followed by AGO2-dependent slicing of the 3p strand, and finally, 3′-5′ trimming of the 5p strand to complete maturation [49].

Once formed, the double-stranded miRNA/miRNA duplex comprises a mature miRNA strand (guide strand) and a complementary miRNA* strand (passenger strand). The mature miRNA or guide strand is then preferentially selected and loaded onto the RNA-induced silencing complex (RISC) and attaches to one of four Argonaute proteins (Argonaute-1, Argonaute-2, Argonaute-3, and Argonaute-4) [50]. The miRNA guides RISC to its target messenger RNA (mRNA), and the miRNA within the RISC binds to target mRNAs through complementary base pairing. This binding can lead to translational repression, mRNA degradation, or a combination of both, depending on the degree of complementarity and other factors [1]. In mammals, mRNA degradation is dependent on Argonaute-2, which possesses enzymatic slicer activity. Alternatively, Argonaute-1, Argonaute-3, and Argonaute-4 inhibit mRNA translation without mRNA degradation. The seed sequence, which is made up of the first 6-8 bases of the miRNA molecule, determine the specificity of miRNA binding with their complementary mRNA sequences [50]. By binding to their target mRNAs, miRNAs regulate gene expression required for various essential biological processes such as normal development, differentiation, metabolism, and immunity. Importantly, miRNAs are pleiotropic; that is, a single miRNA may target numerous mRNAs. Furthermore, a single mRNA may be regulated by several miRNAs. In this way, miRNAs and their target mRNAs form complex regulatory networks that must be carefully deciphered when determining the specific roles and targets of miRNAs [1].

## 6. Innate and Adaptive Immune Cells and Pathways Mediated by miRNAs

As key players in host immunity, miRNAs regulate the survival and functioning of host innate and adaptive immune cells, as well as the signalling pathways involved in host immune responses to infections [35,51]. In innate immunity, miRNAs form regulatory networks and mediate the functions of innate immune cells including granulocytes [52], macrophages [53], dendritic cells [54], and natural killer cells [55]. In adaptive immunity, miRNAs regulate the development, differentiation, selection, and function of T-cells [56] and B-cells [57]. Several miRNAs, such as miR-10a, miR-17-92 cluster, miR-21, miR-29, miR-145, miR-146a, miR-155, miR-181, and miR-223, play key roles in the maturation, proliferation, differentiation, and activation of various innate and adaptive immune cells [comprehensively reviewed by [35,51]].

## 7. Immune Cells and Pathways Mediated by miRNAs in Soil-Transmitted Helminth Infections

Animal models of STHs are widely studied primarily because of their similarities in life cycle to their human counterparts [58]. Animal models such as *Nippostrongylus brasiliensis*, *Heligmosomoides polygyrus, Ascaris suum, Trichinella spiralis,* and *Trichuris muris* have been useful in elucidating important aspects of host–STH interactions and host immunity. In this section of the article, we will provide an overview of the host- and helminth-derived miRNAs reported in STH infections using animal models, with an emphasis on their roles in modulating immune responses. While we have primarily focussed on STH infections, in some instances, we have also reported on miRNAs isolated from other helminth species known to cause human infections.

### 7.1. Immune Cells and Pathways Mediated by Host-Derived miRNAs in Soil-Transmitted Helminth Infections

The role of host miRNAs in regulating crucial components of anti-STH immunity has been recently described [50]. For example, miRNAs regulate the biology and/or function of intestinal epithelial cells, innate lymphoid cells (ILCs), Th2 cells, and regulatory T (Treg) cells during anti-STH immunity: (i) The intestinal epithelial barrier forms an important interface for host–helminth interaction by serving as a physical barrier, detecting parasites and mounting initial host immune responses. Epithelial cells also differentiate into secretory goblet cells which produce increased amounts of mucous during STH infection to facilitate parasite expulsion [50]. MiRNAs have been shown to support the integrity and function of intestinal epithelial cells during anti-STH immunity. Biton and colleagues (2011) reported that mice deficient of the enzyme Dicer were more susceptible to *Trichuris muris* infection than their wildtype counterparts. Dicer deletion compromised the epithelial barrier and led to decreased goblet cell production and reduced expression of resistin-like molecule beta (RELMβ), a Th2 antiparasitic cytokine. The authors also showed that inhibition of miR-375 prevented goblet cell differentiation and TSLP induction in response to IL-13 [59]. (ii) Innate lymphoid cells (ILC2) play essential roles in anti-STH immunity, mainly by producing Th2 cytokines IL-5, IL-9, and IL-13 [24]. MiR-155 regulates appropriate responses by ILC2. In mice infected with *Nippostrongylus brasiliensis*, miR-155 was upregulated in ILC2, while miR-155-deficient mice displayed impaired IL-33-driven ILC2 responses. In vitro, miR-155 promoted ILC2 survival by protecting ILC2 from apoptosis [60]. (iii) Similarly, Okoye and colleagues (2014) [61] demonstrated that miR-155 and miR-146a regulate Th2 cells during anti-STH immunity. Th2 cells drive Th2 responses by releasing key cytokines IL-4, IL-5, IL-9, and IL-13 [24]. Several miRNAs regulate the normal development of Th2 cells, including miR-19a, miR-24, and miR-27 [62]. In the context of anti-STH immunity, T-cell-intrinsic miR-155 promoted Th2 immunity to *Heligmosomoides polygyrus*, by decreasing Sphingosine-1-phosphate-receptor 1 gene (*S1pr1*) expression, which supports the egress of lymphocytes from lymphoid tissue. In contrast, miR-146a inhibited Th2 immunity. T-cell-intrinsic miR-146a prevented overt Th1/Th17 skewing by regulating Th1- and Th17-cell differentiation. The deletion of miR-146a in T-cells was associated with a mixed Th1/Th2/Th17 response and enhanced susceptibility to *Trichuris muris* or *Heligmosomoides polygyrus* infection [61]. More recently, Entwistle and colleagues used a murine model of *Heligmosomoides polygyrus* to study the miRNAome of the intestine during acute reactivation of Th2 immune responses. The authors identified several upregulated host miRNAs, including miR-99a-5p, miR-155-5p, and miR-148a-3p, during active worm expulsion. In addition, miR-99a-5p and miR-155-5p levels were increased in the lungs of mice following allergen-induced acute exacerbation. While pharmacological inhibition of these miRNAs, either individually or in combination, did not compromise antihelminth immunity, the blocking of miR-99a-5p or miR-155-5p was associated with a significant reduction in allergen-induced exacerbation in the lungs [63]. (iv) STH infection induces the production of FoxP3-expressing Treg cells, which modulate host immunity via the production of anti-inflammatory cytokines, IL-10, and TGFβ. Thereby, Tregs reduce STH-mediated inflammation and pathology, creating a conducive environment for STH survival [24]. MiRNAs regulate important aspects of Treg differentiation, function, and survival. MiRNAs such as miR-21, miR-31, miR-24, and miR-210 are known to regulate FoxP3 expression and Treg development [64,65]. In addition, FoxP3 activates miR-155 and miR-146a, by suppressing the suppressor of cytokine signalling-1 (SOCS1) and the signal transducer and activator of transcription 1 (Stat1) effector pathways, respectively [66,67]. In the context of antihelminth immunity, following infection with the trematode, *Schistosoma mansoni*, miR-182 expression was required for Tregs to suppress Th2-associated inflammation, through the regulation of transcription factor *Cmaf* [68]. More recently, it was reported that *Ascaris suum* infection modulated host-derived extracellular vesicle (EV) miRNA expression profiles; the differentially expressed miRNAs were associated with immune-related cells, including Tregs, B cells, and dendritic cells [69].

### 7.2. Immune Cells and Pathways Mediated by Helminth-Derived miRNAs in Soil-Transmitted Helminth Infections

The development of high-throughput technologies, such as next-generation sequencing and microarray profiling, have enabled the discovery of novel helminth-derived miRNAs. To date, miRNAs have been identified in at least 35 helminth species, including nematodes, trematodes, and cestodes of clinical and veterinary importance [70]. Comparative studies show that some miRNAs are conserved across diverse organisms, while others are helminth-specific and novel [71]. To date, helminth-specific miRNAs from various helminth species have been identified in infected hosts, including *Schistosoma japonicum* [72,73], *Schistosoma mansoni* [74], *Onchocerca volvulus* [75,76], *Loa loa* [77], *Onchocerca ochengi* [76,77], *Nippostrongylus brasiliensis* [43], *Heligmosomoides polygyrus* [38], *Necator americanus* [41], *Ascaris lumbricoides* and *Ascaris suum* [39], *Trichuris suis* [40], and *Trichuris muris* [42]. Studies have also shown that the expression patterns of helminth-derived miRNAs differ during developmental stages, between sexes, and within specific cells and tissues [78,79,80,81].

Extracellular vesicles (EVs) are recognized as crucial contributors to cell–cell communication during host–parasite communication and participate in immunomodulatory functions. EVs are membrane-bound bodies secreted by various parasites, including helminths, that contain a repertoire of proteins, lipids, and RNAs, including miRNAs [82]. EVs and their miRNA cargos have been characterized in nematodes, cestodes, and trematodes [reviewed by [82,83,84]. Recent reviews by others have highlighted the role of helminth-derived EVs and their miRNA cargos in modulating host immunity [82,85,86,87]. In support of this, several research groups have studied the immunomodulatory capabilities of helminth-derived EVs [43,88,89]. For example, a previous animal study reported that EVs secreted by the intestinal nematode, *Heligmosomoides polygyrus,* suppressed macrophage activation by downregulating Th1- and Th2-associated molecules and inhibited IL-33 alarmin receptor subunit ST2. Moreover, following vaccination with the helminth EVs, mice developed strong antibody responses, which conferred protection against infection [88]. In another study, *Nippostrongylus brasiliensis*-derived EVs, but not *Trichuris muris*-derived EVs, were shown to reduce gut inflammation in EV-treated mice. In mice treated with *Nippostrongylus brasiliensis*-EVs, reduced gut inflammation was associated with decreased levels of proinflammatory cytokines and increased levels of IL-10 [43]. It has been shown that *Ascaris suum* EVs inhibited tumour necrosis factor (TNF-α) levels in lipopolysaccharide-stimulated peripheral blood mononuclear cells (PBMCs), compared to nonvesicle fractions, which showed no inhibitory activity [89].

An important component of helminth EVs is miRNAs. Studies have demonstrated a role for EV-associated miRNAs in host–parasite interactions and their roles in host innate and adaptive immunity during helminth infection [38,90,91,92,93,94,95]. For example, Buck et al. (2014) demonstrated that synthetic mimics of *Heligmosomoides polygyrus* miRNAs not only shared sequence similarities to host miRNAs but were able to modulate cytokine production in host mammalian epithelial cells. EV-derived miRNAs from the trematode, *Fasciola hepatica*, have been characterized and associated with potential immunomodulatory functions [90,91]. Similarly, during infection with *Fasciola hepatica*, fhe-miR-125b, a conserved miRNA, was incorporated into a host Argonaut protein (Ago-2) within macrophages and negatively regulated the production of inflammatory cytokines [95]. In other studies, EV-derived miRNAs with potential host immune targets, such as IL-13, IL-25, and IL-33, have been reported in *Ascaris suum* [92]. The in vitro uptake of *Schistosoma japonicum* EVs, containing miR-125b and bantam, led to increased macrophage proliferation and TNF-α production through the regulation of their respective targets *Pros1, Fam212b, and Clmp* [93]. In vitro, *Schistosoma mansoni* EVs containing miR-10 regulated MAP3K7 and NF-κB activity and led to impaired Th2 differentiation [94]. The same authors identified helminth-specific miRNAs within the Peyer’s patches and mesenteric lymph nodes of *Schistosoma mansoni*-infected mice [94].

Taken together, these studies highlight molecular mechanisms by which helminths exploit host cells and their miRNA machinery, revealing an important aspect of cross-species communication used by helminths to modulate host immunity.

## 8. Immune Cells and Pathways Mediated by miRNAs in HSV-2 Infections

In this section of the article, we provide an overview of the HSV-2- and host-derived miRNAs reported in HSV-2 infections, with an emphasis on their roles in modulating immune responses.

### 8.1. Immune Cells and Pathways Mediated by HSV-2-Derived miRNAs in HSV-2 Infection

Similarly, many human DNA viruses encode and express unique viral miRNAs (vmiRNAs). Among these are alpha-herpesviruses [Herpes Simplex Virus type 1 (HSV-1) and HSV-2], beta-herpesviruses [human cytomegalovirus (HCMV)], and gamma-herpesviruses [Epstein Barr Virus (EBV) and human herpesvirus 8 (HHV-8)] [96]. In HSV-2, 18 pri-miRNAs and 24 mature miRNAs have been reported. HSV-1 and HSV-2 are closely related, and several miRNAs are conserved between the viruses, bearing more that 70% similarity between their seed sequences [46,47]. VmiRNAs participate in host–viral interactions by regulating host biological functions including cell proliferation, differentiation, apoptosis, and the cell cycle. Thereby, viruses establish infection and produce viral progeny. In addition, vmiRNAs have been reported to contribute to the establishment of latent infection either by regulating metabolic processes or immune responses of host cells [reviewed in [96,97,98,99]]. VmiRNAs can function as orthologs of host miRNAs, thereby regulating their expression. Moreover, vmiRNAs have developed mechanisms to avoid detection by the host immune system [32].

Interactions between HSV-2 vmiRNAs, host miRNAs, and/or target mRNAs can influence HSV-2 replication, immune evasion, latency, and pathology. A characteristic feature of HSV-2 is its ability to establish latent infection in its host. During latency, only the latency-associated transcript (LAT) is expressed. HSV-2 vmiRNAs located within the LAT include miR-H2, miR-H3, miR-H4, miR-H7, miR-H9, miR-H10, and miR-H24 [44,45,47,100]. Studies have shown that, to regulate latency and promote immune evasion, (i) HSV-2-miR-H2 targets *ICP0*, a gene involved in viral replication, lytic infection, and reactivation, and (ii) HSV-2-miR-H1, HSV-2-miR-H3, and HSV-2-miR-H4 target the lytic neurovirulence factor *ICP34.5* [44,45]. In contrast, HSV2-miR-H6, which is associated with HSV2-LAT, was shown to play a role in reactivation of the virus from latency [48]. Taken together, these findings suggest that HSV-2 LAT-encoded vmiRNAs contribute to viral latency and reactivation by regulating their viral targets.

### 8.2. Immune Cells and Pathways Mediated by Host-Derived miRNAs in HSV-2 Infection

In addition to HSV-2 vmiRNAs, host miRNAs (also known as cellular miRNAs) contribute to the establishment of initial infection, reactivation, and viral latency [reviewed in [96,98,99]]. As highlighted earlier, the TLR signalling pathway is initiated during primary HSV-2 infection and is crucial for effective innate and adaptive responses. Host miRNAs have been shown to regulate the TLR pathway during HSV-2 infection and contribute to HSV-2 pathology [101]. Following HSV-2 infection of guinea pigs, six miRNAs (miR-592, miR-1245b-5p, miR-150, miR-342-5p, miR-1245b-3p, and miR-124) were upregulated and correlated with the downregulation of key TLR-associated genes [101]. In another study, host miR-36 was shown to regulate infection of haemopoietic and endothelial cells with the closely related herpesviruses: Kaposi’s sarcoma-associated herpesvirus (KSHV), EBV, and HSV-2 [102]. Host miRNAs have also been reported to suppress viral replication and promote latency of HSV-2. For example, host miR-138 targeted host mRNAs *OCT-1*, *FOXC1*, as well as HSV-2 *ICP0, UL19*, and *UL20* genes, demonstrating that HSV-2 exploits host neuron-specific miRNAs to suppress lytic genes and favour latency [100]. More recently, it was reported that HSV-2 infection of THP-1 macrophages led to the upregulation of immune-associated miRNAs (miR-374a, miR-29b, miR-195, and miR-181a) and the downregulation of their corresponding mRNA targets (IL-10, AKT1, P13K, and AKT2). Suppression of these mRNA targets subsequently led to their inability to inhibit autophagy. In addition, the authors showed that combinatorial miRNA exposure of the THP-1 macrophages prior to HSV-2 infection reduced viral replication and the release of new virions [103].

In summary, host- and pathogen-derived miRNAs are critical regulators of immune responses to helminth and HSV-2 infections, regulating the activation, differentiation, and functions of innate and adaptive cells and signalling pathways of immunity. Therefore, miRNAs contribute significantly to the complexity of host–pathogen interactions, and insight into their mechanisms are required to fully understand these host–pathogen interactions.

## 9. Soil-Transmitted Helminth Immunomodulation of Respiratory, Enteric, and Sexually Transmitted Viral Infections

Experimental and epidemiological studies have shown that STH-induced immunomodulation may influence immune responses to enteric [104,105], respiratory [106,107,108], and sexually transmitted viral infections [9,10,11,13,14,109,110] with varying outcomes. STH co-infection also modulates host immunity to major global pathogenic bacteria and protozoans, which is reviewed elsewhere [6,8]. Here, we provide a brief overview of STH-induced modulation of pathogenic viruses. In the context of STH and enteric viral co-infections, *Heligmosomoides polygyrus* promoted susceptibility to the West Nile virus (WNV) and associated with enhanced intestinal pathology, translocation of intestinal bacteria, and impaired CD8+ T cell responses [105]. Similarly, *Trichinella spiralis* promoted infection with an enteric murine norovirus and associated with alterations in intestinal microbiota and STAT-6-dependent activation of alternative macrophages [104].

In contrast, STH co-infections are beneficial to the outcomes of some respiratory viruses. For example, in mice co-infected with *Heligmosomoides polygyrus,* there was reduced lung disease and inflammation following respiratory syncytial virus [107]. *Trichinella spiralis* reduced influenza virus-induced lung inflammation and associated with lower levels of tumour necrosis factor (TNF) and reduced cellular recruitment in mice co-infected with influenza A virus [106]. More recently, co-infection with *Schistosoma mansoni* or *Trichuris trichiura* was associated with reduced COVID-19 severity in patients infected with SARS-CoV-2 [108].

STHs may also influence the outcomes of sexually transmitted viral infections. Individuals co-infected with STHs and HIV had lower CD4^+^ counts [109], dysregulated immune cells and higher HIV viral loads [11], and altered Th1/Th17 cytokine gene expression [13]. Similarly, hookworm infections increased the risk of HPV infection [110] and correlated with higher HPV viral loads and distinct mixed Type 1/Type 2 vaginal cytokine profiles in women co-infected with hookworm and HPV [10]. Conversely, in vitro infection of cervical cells with *Nippostrongylus brasiliensis* led to reductions in the uptake of HPV16 pseudo-virions, cell migration, and expression of vimentin and N-cadherin [111]. Following infection with the parasite in vivo, there was reduced vimentin expression in the murine vaginal tract, suggesting the parasite’s ability to impair cervical cancer progression [111]. As highlighted earlier, Chetty and colleagues (2021) demonstrated that acute infection of mice with *Nippostrongylus brasiliensis* correlated with enhanced vaginal ulceration following subsequent HSV-2 infection, suggesting that the parasite impairs anti-HSV-2 responses required to adequately control infection. Notably, the authors demonstrated that although the STH neither enters nor resides in the female genital tract, it induced a potent Th2 immune response specific to the female genital tract that altered vaginal immune responses to HSV-2 [14]. Although STH co-infections could be linked to reduced immunity to HSV-2 in endemic regions, there is a significant paucity of data on STH/HSV-2 co-infections. Further studies on HSV-2 pathology in STH-endemic regions are needed to fully elucidate the clinical and public health implications of STH/HSV-2 co-infections.

## 10. Potential Role of miRNAs in Mediating STH/HSV-2 Co-Infections

Looking ahead, the findings by Chetty and colleagues [14] raise several questions regarding the mechanisms by which STH induce a Th2 phenotype in the female genital tract. Furthermore, while there is no definitive evidence regarding STH/HSV-2 co-infections in humans, their findings are suggestive of significant interactions between STHs and HSV-2 and provide a basis for future work. Because STHs and HSV-2 disproportionately affect vulnerable and marginalized communities, it is important to understand how STHs may impact HSV-2 severity. It would be of interest to examine the miRNA expression profile during STH/HSV-2 co-infection and how miRNAs contribute to host immunity during co-infection. To our knowledge, no studies have explored the miRNA profiles during STH/HSV-2 co-infection. However, having described the role that miRNAs play as mediators of immunological responses during single STH and HSV-2 infections, it follows that miRNAs would contribute to the host immune response during STH/HSV-2 co-infection. Although speculative, we contend that miRNA regulation could potentially occur via different mechanisms:

(i) Modulation of host immune responses: As discussed earlier, STH and HSV-2 infections elicit opposing immune responses. HSV-2 requires a robust Th1 immune response for effective viral control [7]. Consequently, host-derived immune-related miRNAs, such as miR-138 [100], miR-36 [102], miR-374a, miR-29b, miR-195, and miR-181a [103], are dysregulated following HSV-2 infection. On the other hand, STHs typically induce Th2-mediated and immunomodulatory responses that suppress Th1-mediated responses [5]. Host-derived miRNAs have been shown to regulate important aspects of anti-STH immunity, including the biology/function of intestinal epithelial cells [e.g., miR-375] [59], ILCs [e.g., miR-155] [60], Th2 cells [e.g., miR-155, miR-146a, miR-19a, miR-24 and miR-27] [62], and Treg cells [e.g., miR-21, miR-31, miR-24, miR-210, miR-182] [64,65,68]. Therefore, in STH/HSV-2 co-infections, several miRNAs could mediate these opposing immune responses. For example, host-derived miRNAs, such as miR-146a and miR-155, are known to regulate inflammation and immune responses, following the recognition of pathogens by TLRs. Specifically, miR-146a regulates the NF-κB pathway by downregulating TRAF6 and IRAK1, which are key adaptors in the TLR signalling pathway. miR-146a also reduces the production of proinflammatory Th1 cytokines, such as TNF-α, IL-6, and IFN-γ [35]. Moreover, studies have shown that the coactivation of miR-146 and miR-155 is regulated by the NF-κB signalling pathway and may contribute to a negative feedback loop, protecting the host against an excessive TLR4 response [53,112,113]. The combined upregulation of miR-146a and miR-155, as previously reported in helminth infections [61,114], may dampen inflammation and reduce anti-HSV-2 responses. In addition, STH-derived EVs and miRNAs may suppress proinflammatory Th1 cytokines, which are critical for HSV-2 control, thereby promoting increased viral replication and persistence. Simultaneously, STH-derived EVs containing miRNAs could modulate key host innate immune signalling pathways, such as the TLR signalling or NF-κB activation to suppress inflammation. This immune suppression could indirectly promote HSV-2 replication and pathology.

(ii) Regulation of viral replication: HSV-2-derived miRNAs, such as HSV-2-miR-H1, HSV-2-miR-H2, HSV-2-miR-H3, and HSV-2-miR-H4, regulate important aspects of viral replication, latency, and reactivation [44,45]. These viral miRNAs might act synergistically with STH-mediated immunomodulation to promote chronic infection. Similarly, certain host-derived miRNAs, such as host miR-138 target viral genes (e.g., HSV-2 *ICP0, UL19*, and *UL20*), limit viral replication [100]. However, during STH/HSV-2 co-infections, STHs may suppress these antiviral miRNAs indirectly by promoting an immunosuppressive environment.

**Figure 2:**
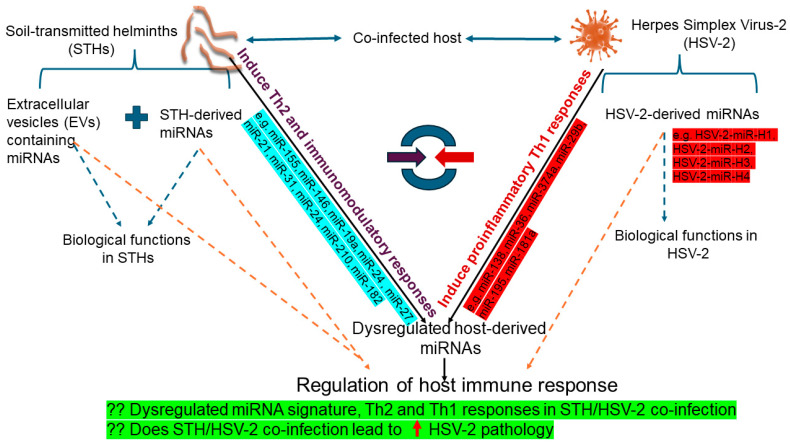
Schematic representation of potential miRNAs involved in regulating STH/HSV-2 co-infection. Host- and pathogen-derived miRNAs have been shown to regulate important biological functions, including aspects of both innate and adaptive immunity, during single STH and HSV-2 infections. Examples of relevant miRNAs are included in the figure. The miRNAs highlighted in blue have been shown to regulate anti-STH immunity, and the miRNAs highlighted in red are known to regulate anti-HSV-2 immunity (as explained in full in text).

In these scenarios, we hypothesise that pathogen-derived and host-derived miRNAs may modulate the host immune response during STH/HSV-2 co-infection, potentially favouring Th2 over Th1 immunity, thus influencing HSV-2 replication, latency, and pathology (Figure 2). Nevertheless, the miRNA profiles and their roles in STH/HSV-2 co-infections remain unexplored, highlighting opportunities for research on STH/HSV-2 co-infections with a particular emphasis on miRNAs.

## 11. Opportunities and Future Directions

As highlighted earlier, helminth-derived miRNAs have been identified in infected hosts and play a role in modulating host immunity and infection. Furthermore, dysregulated miRNA expression is a hallmark of various infections and disease processes [1]. Given the emerging role of miRNAs as diagnostic and prognostic biomarkers, this creates a new avenue for exploration. Specifically, circulating miRNAs possess several attractive features that make them potential diagnostic or prognostic biomarker candidates: (i) They have a broad spectrum of mRNA targets, whereby a single miRNA can regulate multiple mRNA targets and their associated cellular pathways. (ii) They are tissue- and disease-specific, expressing signature patterns specific to a tissue or disease context. (iii) They are highly stable and can withstand freeze-thaw cycles and enzymatic degradation. (iv) They can be detected noninvasively in a range of body fluids such as blood, urine, and saliva [1].

In addition to their diagnostic potential, miRNAs can be leveraged for potential therapeutic purposes. MiRNAs are appealing therapeutic tools because of the ability of a single miRNA to regulate a wide range of mRNA targets and cellular pathways. Briefly, the goal of miRNA-based therapeutics is to reverse dysregulated miRNA expression profiles that are associated with disease processes. MiRNAs and the cellular pathways associated with diseases can be manipulated, by either enhancing or restoring the levels of endogenous miRNAs needed to suppress disease or reducing or inhibiting the function of miRNAs that promote disease. To achieve this, nucleic acids including synthetic miRNAs (miRNA mimics), recombinant vectors with miRNA-encoding sequences, and oligonucleotide-based inhibitors (anti-miRs/miRNA inhibitors) may be used [115]. However, the field of miRNA-based therapeutics is still in its infancy, and several challenges must still be resolved before its successful implementation. Such challenges include identifying the correct administration routes, ensuring in-body stability, targeting the appropriate tissue and cell types, and achieving the overall intended intracellular effects [115].

Given their features such as small size, high sequence similarity, and at times low expression, highly sensitive and specific techniques are required to identify and quantify miRNAs. Methods to detect and study miRNAs such as hybridization (northern blot, microarrays, bead-based profiling), amplification (RT-qPCR), next-generation sequencing, and enzyme-based methods have been recently reviewed [116]. Importantly, miRNAs are embedded within complex regulatory networks and deciphering their specific roles may be challenging. To overcome this, the use of next-generation sequencing techniques and bioinformatics tools to analyse miRNA and mRNA datasets can help unravel these complex networks [117]. Finally, these techniques can be leveraged to transform our understanding of host immunity to STH/HSV-2 single and co-infections. Moreover, elucidating the roles of miRNAs in STH/HSV-2 co-infection may help uncover essential genes and pathways involved in mediating effective host immunity. This could provide novel diagnostic or prognostic biomarkers of infection. Exploring the molecular and immunological basis of STH/HSV-2 co-infections may also uncover novel mechanisms that can be targeted for therapeutic purposes.

## 12. Conclusions

STHs and HSV-2 infections disproportionally affect under-resourced countries, thus representing significant public health challenges. Exploring potential STH/HSV-2 co-infections in these settings is necessary to mitigate the potential negative impact of co-infections. MiRNAs are critical mediators of host immune responses to pathogens. Moreover, they may be exploited by pathogens to enhance pathogen function and survival. Given that miRNAs mediate host responses to STH and HSV-2 infections, it follows that miRNA dysregulation could contribute to susceptibility to infection and subsequent pathology. However, miRNA expression associated with STH/HSV-2 co-infection and their underlying mechanisms are largely unknown. Studies relating to miRNA expression during STH/HSV-2 co-infection are now needed to understand (i) the molecular mechanisms that underpin STH/HSV-2 co-infections and (ii) how dysregulation of miRNAs may contribute to the course of infection. Understanding the roles of miRNAs in host immune responses to STH/HSV-2 co-infection could help in identifying essential genes and pathways involved in facilitating effective host immunity. However, miRNAs are pleiotropic, and they are embedded within complex regulatory networks. Therefore, deciphering their specific roles may be challenging. Techniques such as high-throughput sequencing and bioinformatics analyses of large miRNA and mRNA datasets may help in unravelling these complex networks. This could provide new diagnostic or prognostic biomarkers or novel therapeutic approaches for these important pathogens.
